# Genome Sequence of a Potential New Benyvirus Isolated from Mango RNA-seq Data

**DOI:** 10.1128/genomeA.01250-16

**Published:** 2016-11-10

**Authors:** Noa Sela, Neta Luria, Mor Yaari, Dov Prusky, Aviv Dombrovsky

**Affiliations:** a Department of Plant Pathology and Weed Research, Institute of Plant Protection, ARO, Volcani Center, Rishon LeZion, Israel; b Department of Postharvest Science of Fresh Produce, Institute of Postharvest and Food Sciences, ARO, Volcani Center, Rishon LeZion, Israel

## Abstract

We report here the sequence of a new benyvirus found through mango cultivar Shelly RNA-seq analysis in Israel. This is the first virus strain reported in mango trees.

## GENOME ANNOUNCEMENT

Mango (*Mangifera indica*) suffers from several diseases at all stages of its life. All parts of the plant—namely, the trunk, branch, twig, leaf, petiole, flower, and fruit—are attacked by a number of pathogens, including fungi, bacteria, and algae, but until now no virus infecting mango has been described in the scientific literature. The complete sequencing of mango transcriptome (1) enabled us to detect all mango genes, as well as a new bipartite virus sequence in the transcriptome of the mango fruits.

Although no viral symptoms could be detected, deferential gene analysis of the mango peel transcriptome revealed a substantial stress within the mango peel and an increased level of expression in genes related to plant immune response to pathogens and viral infection (1). *De novo* assembly of the RNA-seq raw data revealed the full sequence of a new full viral genome belonging to the order of positive-strand ssRNA viruses and the genus *Benyvirus*. The draft genome sequence of the virus was determined by *de novo* assembly of a single-end Illumina HiSeq 2000 sequence deposited in the NCBI Sequence Read Archive (SRA) under accession number SRX375390. A new transcriptome was assembled from the 8.6-Gb sequences using Trinity software (2), generating 57,544 contigs with an *N*_50_ of 1,598 bp. In the mango transcript we could find two full contigs of lengths 6,725 bp for RNA1 and 2,516 bp for RNA2. The sequences of the new virus tentatively named *Mangifera indica latent virus* (MILV) were subsequently deposited in GenBank under the accession numbers KU140662 and KU140663, respectively. The RNA1 transcript included only one protein of 2,052 amino acids, and RNA2 contains three different proteins: a putative helicase protein with a viral helicase-conserved domain that has a length of 352 amino acids, a putative coat protein with a tobamovirus coat-conserved domain with a length of 182 amino acids, and a putative movement protein with a movement-protein-conserved viral domain and length of 134 amino acids. The RNA1 long-replicase protein has a sequence homology of 39% to the RNA1-encoding replicase of beet necrotic yellow vein virus (3), 38% to the *Beet soil-borne mosaic virus* (4), 38% to the *Rice stripe necrosis virus* (5), and 39% to the *Burdock mottle virus* (6). Although viruses belonging to the *Benyvirus* genus are the cause of very violent crop diseases, the homolog virus of mango did not show any pathological symptoms on fruits or leaves. We validated the existence of the putative virus MILV by reverse transcription PCR in our mango cDNA library using specific primers designed for the MILV genome.

### Accession number(s).

The whole-genome sequences of *Mangifera indica* latent virus (MILV) RNA1 and RNA2 have been deposited in GenBank under the accession numbers KU140662 and KU140663, respectively.
